# Novel Insights Into the Genetic Causes of Short Stature in Children

**DOI:** 10.17925/EE.2022.18.1.49

**Published:** 2022-05-25

**Authors:** Concetta Mastromauro, Francesco Chiarelli

**Affiliations:** Department of Paediatrics, University of Chieti, Chieti, Italy

**Keywords:** Short stature, children, genetic cause, growth hormone

## Abstract

Short stature is a common reason for consulting a growth specialist during childhood. Normal height is a polygenic trait involving a complex interaction between hormonal, nutritional and psychosocial components. Genetic factors are becoming very important in the understanding of short stature. After exclusion of the most frequent causes of growth failure, clinicians need to evaluate whether a genetic cause might be taken into consideration. In fact, genetic causes of short stature are probably misdiagnosed during clinical practice and the underlying cause of short stature frequently remains unknown, thus classifying children as having idiopathic short stature (ISS). However, over the past decade, novel genetic techniques have led to the discovery of novel genes associated with linear growth and thus to the ability to define new possible aetiologies of short stature. In fact, thanks to the newer genetic advances, it is possible to properly re-classify about 25–40% of children previously diagnosed with ISS. The purpose of this article is to describe the main monogenic causes of short stature, which, thanks to advances in molecular genetics, are assuming an increasingly important role in the clinical approach to short children.

Short stature affects 2.5% of children and is one of the most common reasons for consulting a growth specialist during childhood.^1^ Normal height is a polygenic trait and derives from the interaction of several factors. It is known that height is influenced by hormonal, nutritional and psychosocial variables, and during recent years it has been increasingly demonstrated that genetic differences have a strong correlation with height.^2^ In fact, around 80–90% of adult height is heritable.^2^ Genome-wide association studies have demonstrated the existence of a large number of genetic loci associated with growth, which explains about 27% of the adult human height variation.^3^ However, the effect of gene mutation on height depends on the type of mutation itself. In fact, polymorphisms and mild mutations could modulate an individual's height, which may remain in the normal range and cause a mild short stature, while more important mutations with effect on protein function, gene haploinsufficiency or biallelic mutations are the basis of isolated monogenic causes of short stature or skeletal dysplasia.^4^

There are multiple causes of short stature, which can be divided into three main groups according to the International Classification of Paediatric Endocrine Diagnoses: 1) primary growth disorders, including genetically based syndromes, small for gestational age with failure of catch-up growth and skeletal dysplasias; 2) secondary growth disorders, which are related to hormonal, nutritional and environmental causes or to specific organ diseases; 3) idiopathic short stature (ISS).^5^ Therefore, among these different forms, ISS is a diagnosis of exclusion. In particular, it defines children with normal birth size (birth weight and length > -2 standard deviation [SD]), normal body proportions and height < -2 SD of the mean height for the age, sex and population, in the absence of identified systemic, endocrine, nutritional or genetic causes.^5,6^ According to the familial recurrence, ISS can be classified into two major categories, namely familial and non-familial short stature. Familial short stature may be characterized by the presence of a height below 2 SD for given age, sex and population but within the parental target range. In contrast, non-familial short stature occurs when the height is both below 2 SD for a given age, sex and population and below the mid-familial height.^7^

In all children, a systematic approach based on clinical and hormonal diagnostic techniques remains important in identifying the underlying cause of short stature. However, in the majority of cases, **short stature is not related to laboratory findings that might affect growth (e.g. chronic disease or hormone dysfunction).**^8,9^ The emergence of genome-wide association tools and novel genetic analysis has paved the way to discovering a wide range of genes related to short stature, which has helped to broaden the possible causes of short stature to be considered during the diagnostic process. The addition of genotyping to the diagnostic approach allows the identification of new causes of short stature and is also useful in understanding the physiology of normal growth. Therefore, the newer genetic techniques help in properly classifying those children still labelled as having ISS. A diagnostic approach that combines clinical, endocrine and genetic assessment is important in establishing a correct aetiology of the disease.^10^

**Figure 1:**
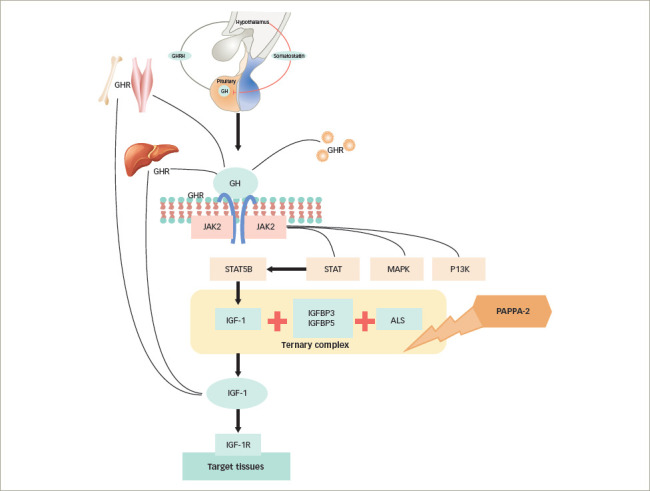
Genetic defects of growth hormone–insulin-like growth factor-1 axis

*GH binds to a GHR, which activates JAK2 and promotes the phosphorylation of several members of the signal transducer and activator of transcription family such as STAT-5b. This process finally results in IGF-1 production. IGF-1 binds to IGFBP-3 or IGFBP-5 and the acid-labile subunit (ALS), encoded by the gene IGFALS, forming the ternary complex. PAPPA-2 is a metalloproteinase that proteolyzes IGFBP-3 and IGFBP-5 increasing the bioavailability of IGF-1.*

*ALS = acid-labile subunit; GH = growth hormone; GHR = GH receptor; IGF-1 = insulin-like growth factor-1; IGF1R = Insulin-like growth factor 1 receptor; IGFBP = IGF binding protein; JAK = Janus kinase; MAPK = mitogen-activated protein kinase; PAPPA-2 = pregnancy-associated plasma protein-2; PI3K = phosphatidylinositol 3-kinases; STAT = signal transducer and activator of transcription.*

The purpose of this article is to describe the main monogenic causes of short stature, which, thanks to advances in molecular genetics, are assuming an increasingly important role in the clinical approach to short children.

## Short stature: Genetic causes

The presence of a phenotype–genotype correlation might normally amplify the diagnostic accuracy in the evaluation of growth failure. However, as the clinical phenotype is often not suggestive of a specific pathology, it is difficult to identify short children who are more likely than others to have a genetic mutation. Genetic mutations can lead to alterations not only of hormonal components, but also of fundamental cellular processes such as intracellular signalling pathways, transcriptional regulation and DNA repair, extracellular matrix or paracrine signalling. Moreover, heterogeneous mutations in one gene can result in different clinical entities; conversely, a single clinical disorder can result from mutations in different genes.

Below, we describe the main and so far most characterized monogenic defects associated with short stature related to anomalies of the growth hormone (GH)–insulin-like growth factor (IGF)-1 axis and the growth plate pathway.^11^

## Genetic defects of the growth hormone–insulinlike growth factor-1 axis

The GH–IGF-1 axis is a fundamental component of normal growth. Anomalies in this pathway commonly lead to GH or IGF-1 deficiency. Nevertheless, mild modifications of GH or IGF-1 levels might be related to different gene defects implicated in the GH–IGF-1 axis. In the past, genetic causes of short stature were mainly associated with defects in the GH–IGF-1 axis. However, recently other genes not involved in the GH–IGF-1 axis have also been associated with monogenic short stature.

As shown in *Figure 1*, the genetic defects can be located at any level on the GH–IGF-1 axis, from the hypothalamus to the tissues' target receptors.^12^ The main genetic mutations might induce three major biological effects that can be summarized into three categories: 1) GH deficiency (GHD); 2) GH insensitivity (GHI) and low activity of IGF-1 and IGF-2; 3) IGF-1 insensitivity (*Table 1*).

**Table 1:** Main genetic defects of the growth hormone–insulin-like growth factor-1 axis and their relevant clinical features

Defect	Gene(s)	Inheritance	Clinical features
GH deficiency	*GH1* (type IA)	AR	Most severe short stature
	*GH1, GHRHR* (type IB)	AR	Less severe short stature
	*GH1* (type II)	AD	Variable short stature
	*BTK, SOX3, GHSR* and other (type III)	X-linked	Short stature, agammaglobulinaemia
GH insensitivity / IGF-1 or IGF-2 deficiency	*GHR* (Laron syndrome)	AR/AD	Severe growth failure Mid-facial hypoplasia
	*STAT5B*	AR	Variable short stature, mid-facial hypoplasia, immunodeficiency, pulmonary fibrosis
	*IGF1 (IGF2)*	AR	Extreme prenatal and postnatal growth impairment, microcephaly, developmental delay, sensorineural deafness
	*IGF-ALS*	AR	Mild growth failure, delayed puberty
	*PAPPA2*	AR	Variable severity of short stature, skeletal abnormalities (very thin long bones, low bone mineral density) insulin resistance, microcephaly
	*IGF2*		Prenatal and postnatal growth failure
IGF-1 insensitivity	IGF1R	AD, AR	Prenatal and postnatal growth failure, microcephaly, developmental delay

*AD = autosomal dominant; ALS = acid-labile subunit; AR = autosomal recessive; BTK = Bruton's agammaglobulinaemia tyrosine kinase; GH = growth hormone; GHR = GH receptor; GHRHR = growth hormone-releasing hormone receptor; GHSR = ghrelin receptor; IGF = insulin-like growth factor; IGF1R = Insulin-like growth factor 1 receptor; PAPPA2 = pregnancy-associated plasma protein A2; SOX3 = sex-determining region Y-related high mobility group-box-3; STAT-5B = signal transducer and activator of transcription-5B.*

### Growth hormone deficiency

Isolated GHD is the most common pituitary hormone deficiency and includes genetic and acquired causes, as well as idiopathic causes due to unknown genetic mutations. Most of these cases will probably be revisited in the very near future following development of novel and advanced techniques, thus allowing a reclassification of GHD types.

According to the study population,^13^ GHD is discovered in about 1:4,000 to 1:10,000 live births.^13^ However, most cases of short stature result from nonpituitary aetiology and are incorrectly classified as GHD.^14^ Among individuals with isolated GHD, between 3% and 30% have familial forms.^12^ The most frequent causes of isolated GHD include mutations affecting the genes for GH (*GH1*) or the GH-releasing hormone receptor (*GHRHR*), leading to GH synthesis and secretion defects. More rarely, GHD results from anomalies of hypothalamic and pituitary development during the embryonic life, which commonly lead to multiple pituitary hormone deficiencies.^15^

Classical GHD includes several types. Type IA is the most severe form of isolated GHD. It is characterized by the complete absence of detectable serum GH due to *GH1* deletion. It is inherited as an autosomal recessive transmission. After GH administration, most patients produce anti-GH antibodies, and thus the height of these individuals does not persistently increase after an initial spurt in response to recombinant human GH (rhGH) therapy; this characteristic may help to confirm the diagnosis. The lack of endogenous production of GH explains the immune response after rhGH. Anti-GH antibodies have various linking affinities and block GH binding to the receptor. Differences in immune tolerance are related to the type of *GH1* mutation and its severity, to the human leukocyte antigen haplotypes, or to other genes of immune response as well as to the age at first antigen contact.^16^ Type IB is less severe. It is characterized by low but detectable levels of GH and it may result from splice site, frameshift, missense or non-sense defects of *GH1* or *GHRHR*. It shows the absence of anti-GH antibodies after treatment. Type II is inherited as an autosomal dominant disease. Many cases are characterized by the production of a cut isoform of GH with a negative impact on formation of the intact GH molecule and potentially of other pituitary hormones. Thus, 5–45% of patients previously categorized as having GHD may develop multiple pituitary hormone deficiency.^17^ The X-linked type III is often related to agammaglobulinaemia, caused by 600 different mutations of the Bruton's agammaglobulinaemia tyrosine kinase gene (*BTK*). Nevertheless, it remains unclear how abnormalities of this protein may cause GHD.^18^ In addition, disorders in pituitary development can also cause GHD. These abnormalities usually lead to combined pituitary hormone deficiencies.^17^ Among these, septo-optic dysplasia, is a syndromic form of congenital hypopituitarism characterized by optic nerve hypoplasia, agenesis of mid-line brain structures and pituitary gland hypoplasia.^19^ The common genetic abnormalities involve two gene variants, namely *HESX1* and *SOX2*.^20^ *HESX1* is a homeobox gene, which acts as a transcriptional repressor and is responsible for pituitary organogenesis.^19^ The *SOX2* gene is important in the development of the pituitary gland, forebrain and eye during human embryogenesis.^21^ Other less common genes involved in the pathogenesis of septo-optic dysplasia are *SOX3* and *OTX2*;^22^ heterozygous mutations of these latter transcription factors involved in pituitary development are other possible causes of isolated GHD.^23^

Another potential defect causing partial GHD, involves the gene encoding for the ghrelin receptor (*GHSR*). The role of *GHSR* mutations in causing short stature is unclear because of the variability of clinical phenotypes and the incomplete segregation of the mutations with the phenotype. However, different studies have proposed that *GHSR* mutation may reduce GH production, thus suggesting the correlation between *GHSR* mutations and short stature.^24^

### Growth hormone insensitivity and insulin-like growth factor-1 low activity/deficiency

Mutation at any level of the GHRH–GH–IGF-1 axis can cause GHI or IGF-1 deficiency, which must be differentiated from GHD by the presence of normal or high serum GH levels.^25^ Consequently, primary IGF-1 deficiency includes: GHI syndrome, genetic anomalies of the GH signalling pathway, defects of IGF-1 bioavailability and deletions or mutations of the *IGF-1* gene itself.

The first cause of GHI is Laron syndrome, caused by a homozygous mutation of the gene encoding the GH receptor (*GHR*), resulting in complete GHI. Individuals with Laron syndrome show low serum IGF-1 concentrations and high levels of circulating GH. GHI is confirmed by the failure to increase IGF-1 or IGFBP-3 concentrations after exogenous GH administration. Frequently, serum GH binding protein (GHBP) is absent, except when the mutation involves the intracellular or transmembrane part of the protein because GHBP is in the extracellular domain of GHR. The classical form is related to important growth restrictions; however, there are milder phenotypes.^26,27^

In addition, abnormalities downstream of GHR can cause the same dysfunction. Among these, mutations of *STAT5B*, which is part of the signalling cascade of the GH receptor, have been well characterized. As GHR does not have intrinsic kinase activity to promote GH action, the engagement of cytosolic Janus kinase-2 and phosphorylation of different transduction factors are needed. These factors are mitogen-activated protein kinase (MAPK), phosphatidylinositol 3-kinases and the transcription family signal transducer and activator of transcription (STAT). Among these, STAT-5 seems to play the most predominant role by activating the transcription of IGF-1. In addition to growth failure, most children with homozygous inactivating *STAT5B* mutations, have an associated immunodeficiency and pulmonary fibrosis.^28^ Moreover, a heterozygote condition for a *STAT5B* mutation has been described.^29^ Scalco et al. showed that heterozygous *STAT5B* mutations have a significant negative impact on height.^29^ However, the height is usually within the normal range, demonstrating a milder effect than that resulting from homozygous mutations. These results suggested that heterozygosity of rare pathogenic variants contributes to normal height heritability.^29^

Homozygous mutations of the *IGF1* gene, causing its complete loss of function, result in extreme prenatal and postnatal growth impairment and are frequently associated with various forms of microcephaly, developmental delay and sensorineural deafness due to the IGF-1 role in nervous system development in utero.^30^ Heterozygous mutations of the *IGF1* gene can cause mild growth failure characterized by lower birth weight, height and head circumference but normal hearing.^31^ Although height SD score (SDS) was significantly lower than in the non-carriers, it was still within the normal range.^31^

Growth restriction may also be a consequence of the alteration of IGF-1 bioavailability. IGF-1 may act locally (autocrine/paracrine), but most frequently it circulates bound as a ternary complex.^15^ In fact, IGF-1 is initially secreted into the blood and subsequently binds to a group of six high-affinity IGF binding proteins (named from IGFBP-1 to IGFBP-6), and among these IGFBP-3 is the most common in adult serum. Soon after, the binary complex is stabilized by the linking to an acid-labile subunit (ALS), encoded by the *IGFALS* gene, thus forming the ternary complex. Most of the serum-circulating IGF-1 (approximately 80%) is included in this complex (*Figure 1*), which increases the serum IGF-1 half-life.^1^ In fact, biallelic loss-of-function mutations in *IGFALS* result in severe reduction in levels of circulating functional *ALS* and the inability to produce a ternary complex, with consequently lower IGF-1 and IGFBP-3 levels. Therefore, children with ALS mutations may have reduced serum IGF-1 and even lower IGFBP-3 concentrations, as well as mild growth failure and delayed puberty.^32^ Heterozygous variants are responsible for isolated short stature.^33^ In fact, heterozygous carriers seem to be shorter than siblings and present lower levels of IGF-1, IGFBP-3 and ALS; delayed puberty is frequently observed in these patients, typically in males.^33^

The release of free IGF-1 from the ternary complex is mediated by pregnancy-associated plasma protein A-2 (PAPPA-2), which is a metalloproteinase that proteolyzes IGFBP-3 and IGFBP-5. Therefore, the bioavailability of IGF-1 in patients with *PAPPA2* mutations is very low. In addition to a variable severity of short stature, children with autosomal recessive mutations in the *PAPPA2* gene have very thin long bones, low bone mineral density, insulin resistance and microcephaly of varying degrees, apparently due to insufficient availability of free IGF-1.^34^

IGF-2, is known to affect both prenatal and postnatal growth, as shown in most children with Silver–Russell syndrome. Imprinting disorders with hypomethylation of genetic control regions such as the *H19* gene may result in down-regulation of the IGF-2 paternally expressed allele, and are related to prenatal and postnatal growth impairment such as in Silver–Russel syndrome and Temple syndrome. These children exhibit partial IGF-1 resistance associated with relatively high IGF-1 and IGFBP-3 levels.^35^ The typical features include prenatal and postnatal growth failure, underweight, relative macrocephaly, triangular face, body asymmetry, and several minor anomalies such as clinodactyly V.^36,37^ Conversely, among imprinting disorders, the most common overgrowth syndrome is represented by Beckwith–Wiedemann syndrome. The 80% of cases result from a known molecular aberration affecting the regulation of a group of imprinted genes related to somatic growth control located in chromosome 11p15.^5,38^ Beckwith–Wiedemann syndrome presents with a variable clinical spectrum represented by macrosomia, macroglossia and abdominal wall defects, and increases the predisposition to cancer.^38^ Among imprinting disorders, another well-known example is Prader–Willi syndrome. The most frequent genetic subtype is related to paternal chromosome 15q11q13 deletion. Typical features are reduced muscle strength, altered body composition, obesity, low energy expenditure, short stature and delayed bone age.^36^ As GH secretion can be reduced, GH treatment has positive effects on linear growth.^39,40^ Indeed, different studies have also postulated a beneficial effect of GH therapy on body composition of these patients.^41^ In fact, a recent Spanish study showed increased lean mass, decreased fat mass and raised neural activation in different cerebellar areas in treated individuals.^42^

### Insulin-like growth factor-1 insensitivity

Defects of the gene encoding IGF-1 (and IGF-2) receptors produce IGF-1 insensitivity. Homozygous or compound heterozygous IGF-1 receptor (*IGF1R*) defects are related to a more severe phenotype. The clinical phenotype is characterized by prenatal and postnatal growth restriction, microcephaly and developmental delay. IGF-1 levels are in the upper half of the normal range and can become very high after GH treatment, because of the decreased sensitivity. In addition, *IGF1R* copy number variants can cause prenatal and postnatal growth restriction or overgrowth.^43^ However, IGF-1 insensitivity may also be related to mutation downstream of the IGF-1 receptor or by defective micro-RNA regulation of IGF-1 signalling.^11,44^ This condition may also be associated with other diseases such as cardiac defects and impaired glucose tolerance.^45,46^

## Mutations of genes involved in the growth plate

Recently, the growth plate has become increasingly important in the study of genetic causes of short stature. Endochondral ossification occurs into the growth plate and promotes bone elongation. It is characterized by proliferation, hypertrophy, and senescence of chondrocyte and cartilage matrix synthesis.^47^ Endochondral ossification is regulated by paracrine and autocrine components. Therefore, mutations in genes that modify this balance have been associated with growth failure. These defects are likely to cause variable body disproportion with different degrees of severity. We discuss the most common alterations of this pathway, classified by Wit et al.^11^ into three groups: 1) genetic defects of growth plate paracrine factors, 2) defects of cartilage extracellular matrix, and 3) genetic anomalies of intracellular pathways (*Table 2*). The definition of these different groups is relevant to properly define some specific peculiarities.

**Table 2:** Main genetic defects affecting the growth plate and their relevant clinical features

Defects	Gene(s)	Inheritance	Clinical features
Genetic defects of growth plate paracrine factors	*FGFR1 FGFR2 FGFR3*	AD	**Achondroplasia**: disproportionate short stature, rhizomelic limb shortening, macrocephaly, variable frontal and parietal bossing, mid-facial hypoplasia, limited elbow extension **Hypochondroplasia**: short stature, generalized laxity, limited elbow extension, short-limbed dwarfism, brachydactyly, relative macrocephaly
	CNP–NPR2 pathway	AR	**Acromesomelic dysplasia** (Maroteaux type): severe skeletal dysplasia, extremely short stature, disproportionate shortening of middle and distal segments
		AD	**Heterozygous**: mild growth impairment, mesomelia
Genetic defects of cartilage extracellular matrix	*ACAN*	AR	Spondyloepimetaphyseal dysplasia aggrecan type: severe short stature, mid-facial hypoplasia, brachydactyly, rhizomelia, mesomelia, facial dysmorphism
		AD	**Spondyloepiphyseal dysplasia Kimberley type**: mild skeletal dysplasia, proportionate short stature, osteoarthropathy or short stature with advanced bone age
Genetic defects of intracellular pathways	*SHOX*	AR	**Langer mesomelic dysplasia**: severe skeletal dysplasia, extreme short stature, mesomelia, Madelung deformity of the wrist
		AD	**Leri–Weill dyschondrosteosis**: variable short stature, milder skeletal dysplasia, mesomelia

*ACAN = aggrecan; AD = autosomal dominant; AR = autosomal recessive; CNP = C-natriuretic peptide; FGFR = fibroblast growth factor receptor; NPR2 = natriuretic peptide receptor 2; SHOX = short stature homeobox.*

### Genetic defects of growth plate paracrine factors

Many genetic mutations of paracrine components are associated with various degrees of skeletal dysplasia.

**Figure 2:**
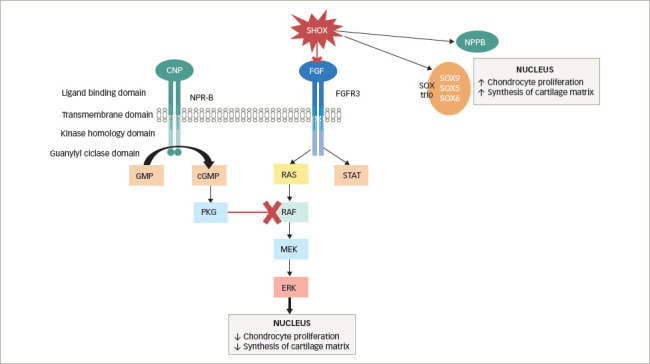
Genetic defects of growth plate paracrine factors

The fibroblast growth factors (FGFs) play a role in the growth plate. Among these, fibroblast growth factor receptor-3 (FGFR-3) negatively regulates the growth plate chondrogenesis. Heterozygous activating mutations in *FGFR3* inhibit skeletal growth. These effects are mainly due to the ability of FGFR-3 to accelerate hypertrophic differentiation. It also decreases proliferation of both the proliferative zone and the matrix.^48^ Therefore, the activation of FGFR-3 suppresses long-bone elongation, and consequently induces skeletal dysplasia (*Figure 2*).

Activating mutations of *FGFR3* lead to a wide range of phenotypes, such as thanatophoric dysplasia, achondroplasia and hypochondroplasia, related to a different mutation site. Achondroplasia is caused by a recurrent defect in the transmembrane domain of the *FGFR3* gene that determines its activation.^49^ Clinical features include long-bone shortening, especially in the proximal region of the upper and lower extremities, resulting in a disproportionate short stature and macrocephaly with variable frontal and parietal bossing and mid-facial retrusion.^50^

Hypochondroplasia is associated with *FGFR3* cytoplasmic (immunoglobulin-like) domain activating mutations. The typical clinical features are short stature, generalized laxity, limitation of elbow extension, rhizomelic limb shortening, brachydactyly and relative macrocephaly.^51^

Another pathway includes C-natriuretic peptide (CNP) and natriuretic peptide receptor B (NPR-B). CNP is encoded by the *NPPC* gene and is a positive regulator of growth plate function. The *NPR2* gene encodes NPR-B, which has a great affinity for CNP. Both CNP and NPR-B are present in the hypertrophic zone of the growth plate. When CNP links to NPR-B, after dimerization of the NPR-B the guanylyl-cyclase is activated and then intracellular cyclic guanosine monophosphate (cGMP) stimulates the type II cGMP-dependent protein kinase I and II (cGKI and cGKII). This activation inhibits the MAPK cascade and consequently decreases FGFR-3 signalling.^39^ Thus, CNP is a negative regulator of FGFR-3 and the process results in the increase of chondrocyte proliferation and differentiation (*Figure 2*).

There is no specific phenotype for CNP–NPR-B-related mutations. Individuals with these mutations present a variable degree of short stature, abnormal body proportion (sitting height:height ratio SDS >2), and different skeletal abnormalities. Homozygous inactivating mutations of *NPR2* result in acromesomelic dysplasia or Maroteaux type, a severe skeletal dysplasia. These individuals present extremely short stature and limbs, with disproportionate shortening of forearms and forelegs (middle segments) and of hands and feet (distal segments).^52^

A heterozygous CNP–NPR-B-related mutation causes mild growth impairment characterized by disproportionate short stature with features similar to those of *SHOX* haploinsufficiency. These individuals exhibit mesomelia but they do not manifest Madelung's deformity.^53^ According to recent studies, heterozygous loss-of-function mutation of the *NPR2* gene is responsible for about 2–3% of ISS cases.^54^ Conversely, heterozygous gain-of-function mutations in *NPR2* are related to variable tall stature.

Although no approved drugs are yet available, promising molecules acting on these pathways are being tested. Among these ‘Vosorotide’ (analogue of CNP) has been tested. It works by increasing the production of extracellular matrix, which, along with chondrocytes, serves as the basis for endochondral ossification.^55^ Another drug that aims to improve anatomical proportions is TA-46. It is a recombinant FGFR-3 ligand trap (a soluble form of human FGFR-3).^55^Other research has tested the efficacy of ‘infigratinib’, a pan-FGFR tyrosine kinase inhibitor that is more selective for FGFR-3 than for other FGFRs. It counteracts the hyperactivity of FGFR-3 by blocking the tyrosine kinase activity mainly of the MAPK receptor (but also of the STAT and sex-determining region Y-related high mobility group-box [SOX]-9 pathways) at the intracellular level.^55^ Further research is needed to determine the role of CNP analogues in growth stimulation in these patients.

*FGFR-3 is a negative regulator of growth plate chondrogenesis. NPR-B activation induces increase of intracellular cGMP and activation of PKG. CNP and FGF pathways converge at the MAPK pathway. The opposite effect of CNP on FGF improves longitudinal bone growth by increasing chondrocyte proliferation and differentiation. SHOX is a transcriptional factor that enhances NPPB and SOX trio and inhibits FGFR3 expression.*

*cGMP = cyclic guanosine monophosphate; CNP = natriuretic c-peptyde; FGF = fibroblast growth factor; FGFR-3 = fibroblast growth factor receptor-3; NPPB = natriuretic peptide B; NPR-B = natriuretic peptide receptor type B; MAPK = mitogen-activated protein kinase; PKG = protein kinase G; SHOX = short stature homeobox; SOX = sex-determining region Y-related high mobility group-box; STAT = signal transducer and activator of transcription.*

### Genetic defects of the cartilage extracellular matrix

Chondrocytes secrete extracellular matrix, composed of collagenous and non-collagenous proteins and proteoglycans, which are essential to assure normal growth plate function. In the cartilage extracellular matrix, one of the most important mutations affects the *ACAN* gene, encoding the cartilage matrix proteoglycan aggrecan (ACAN). The *ACAN* mutation reduces chondrocyte proliferation and accelerates hypertrophic chondrocyte differentiation, producing abnormal cartilage extracellular matrix. Defects in the *ACAN* gene are recognized in approximately 1.5% of patients with ISS.^56^ These individuals have a compromised adult height with lower adult height SDS and body disproportion due to the insufficient pubertal spurt followed by precocious growth interruption.^57^ Homozygous defects cause a severe skeletal dysplasia, known as spondyloepimetaphyseal dysplasia aggrecan type, characterized by severe short stature, mid-face hypoplasia and brachydactyly.^58^ Heterozygous defects can cause spondyloepiphyseal dysplasia Kimberley type, which is a mild form of skeletal dysplasia characterized by proportionate short stature and progressive osteoarthropathy, usually affecting the knees with variable severity due to the ACAN role in the articular cartilage. In addition, most children have proportionate short stature in the presence of advanced bone age without evident radiographic skeletal dysplasia,^59^ although advanced bone age might not be detected in all cases.

Indeed, recent studies have focused on collagen diseases as a possible cause of growth failure.^60^ In the growth plate, collagen types II, IX, X and XI are present; among them type II collagen represents the most abundant component. Collagenopathies type II are related to *COL2A1* variants and are associated with short stature and skeletal dysplasia, although there is not a clear genotype–phenotype correlation.^60^ However, future studies are needed to better characterize the relationship between the different components of the growth plate and the causes of growth impairment.

### Genetic defects of intracellular pathways

The most significant defect of this group is the aberration of the gene encoding short stature homeobox (*SHOX*). This gene is a part of the sexual chromosomes X and Y, particularly at pseudoautosomal region 1 (PAR1). *SHOX* is a transcriptional activator expressed in hypertrophic chondrocytes. It stimulates and coordinates chondrocyte proliferation and differentiation through activation of NPPB and inhibition of *FGFR3* expression, thus promoting longitudinal growth. Moreover, *SHOX* interacts with the SOX trio (*SOX9*, *SOX5*, *SOX6* genes), which is involved in cartilage matrix production (*Figure 2*). *SHOX* defects affect about 2–15% of children classified as ISS, depending on the study and the selection of children.^61^ The function of the *SHOX* gene is dose dependent: the loss-of-function mutation of a *SHOX* allele (haploinsufficiency) causes SHOX deficiency associated with growth failure. The majority of *SHOX* mutations, approximately 80%, are deletions of different sizes including the *SHOX* gene itself or the enhancer region downstream of the coding region,^62^ the remaining ones being missense and nonsense mutations that are spread throughout the gene. These mutations are expected to cause protein inactivation or block nuclear translocation or dimerization of SHOX.

The main pathological conditions related to haploinsufficiency of the *SHOX* gene range from Langer mesomelic dysplasia, Leri–Weill dyschondrosteosis (LWD) to isolated short stature.^63^

Langer mesomelic dysplasia is the most severe and a rare condition caused by homozygous or compound heterozygous inactivating *SHOX* defects. This condition is characterized by critical skeletal dysplasia, extreme short stature, with short long bones of the arms and legs such as ulna and fibula (mesomelia), as well as Madelung deformity of the wrist.^64,65^ Madelung deformity is due to anatomical modifications of the wrist consisting of shortening and curving of the radius, prominence of the ulnar head, and palmar and ulnar deviation of the carpal bones.^66^ Radiologically, Madelung deformity is diagnosed by the absence or narrowing of the ulnar portion of the distal radial physis, anterior curvature of the radial shaft and dorsal subluxation of the ulnar head.^67^ Histopathological analysis shows the chondrocytes in the growth plate having an altered distribution, characterized by a side-by-side arrangement.^68^

In addition to wrist deformity, there are other less specific signs of SHOX deficiency. These features include: shortening of the fourth and fifth metacarpals, arched palate, increased transport angle of the elbow, scoliosis and micrognathia.^69^ One-third of individuals may also have calf muscle hypertrophy.^69^ The absence of one of these signs does not exclude *SHOX* haploinsufficiency.^61^

Heterozygous mutations cause LWD and involve about 2.6–12% of cases of ISS.^39^ LWD is characterized by a variable degree of short stature and milder skeletal dysplasia.^70^ SHOX is also involved in growth failure documented in individuals with Turner syndrome when the mutation of the X chromosome includes PAR1.^71^ However, even heterozygous deletions of the downstream and upstream enhancer of *SHOX* are related to the same phenotype of the *SHOX* gene defect itself.^72^ A clinical rule has been developed to identify children with *SHOX* defects that are suitable for genetic analysis. The most frequently used parameters include the extremities: trunk ratio, the sitting height:height ratio or other scoring systems including different anthropometric measurement and dysmorphic features.^69^ However, these systems are limited by the high variability in the phenotype of SHOX deficiency.

Another intracellular growth plate-related pathway is the Ras–MAPK signalling pathway, comprising different genes such as *PTPN11*, *SOS1*, *RAF1*, *KRAS*, *BRAF* and *NRAS*. Mutations of this pathway result in a group of syndromes called ‘rasopathies’, including Noonan syndrome.^73^ These syndromes are characterized by a variable degree of growth impairment that can be associated with other phenotypic features such as facial dysmorphism and a large spectrum of congenital heart defects in cases of Noonan syndrome.^74^

## Diagnostic approach

Before assuming a genetic anomaly, it is important to follow an appropriate diagnostic approach that allows the clinician to exclude the most frequent causes of short stature. The early identification of children with abnormal growth requires an efficient growth-monitoring system, accurate auxological characterization and appropriate diagnostic work-up in order to identify patients who require closer examination.^75,76^

In fact, the physical examination is important for detection of characteristic features related to the different causes of short stature. For example, Turner syndrome and presumably also *SHOX*, *NPR2* and *ACAN* defects present a typical growth curve characterized by a low–normal birth length, declining length SDS for 2 years, static height SDS in childhood, and further height SDS reduction during adolescence.^77^ The presence of disproportion should be evaluated by assessing sitting height, arm span and sitting height:height ratio. Abnormal body proportions are strongly indicative of skeletal dysplasia. The presence of dysmorphic features should be evaluated to determine a proper diagnosis. In particular, thin long bones associated with microcephaly are related to *PAPPA2* mutations, while macrocephaly is characteristic of mutations activating FGFR-3. The presence of microcephaly is also associated with GHI defects such as IGF-1 and IGF-2 defects and *IGFALS* and *IGF1R* mutations. Similarly, mesomelia and Madelung deformity are suggestive of SHOX deficiency.^76^ The presence of microcephaly is suggestive of IGF-1-related genetic abnormalities. The examination of facial and body dysmorphic features is important for detection of syndromic diseases in which short stature is one of the associated features.^78^

Laboratory tests should be guided by clinical features rather than routinely applied to all patients with short stature. To identify the aetiology of short stature, primary and secondary growth disorders first need to be excluded. According to the 2008 ISS consensus statement,^5^ the common panel for laboratory tests includes full blood count, thyroid function, IGF-1, IGFBP-3, screening for coeliac disease, electrolyte, renal and liver function, calcium-phosphorus metabolism and karyotype in girls. However, Collett-Solberg et al. have since suggested that laboratory analysis should be performed on a case-by-case basis, led by clinical characteristics.^76^ Therefore, if the clinical assessment is indicative of a specific disease, targeted laboratory investigations should be conducted to determine the underlying pathology.^78^ In this phase of the diagnostic process, the study of the GH axis is of considerable importance in order to exclude a potential defect in its function. The consensus guidelines suggest the measurement of GH-dependent factors (IGF-1 and IGFBP-3). If serum IGF-1 is lower than normal, a GH stimulation test measuring GH levels after pharmacological stimulus or an IGF generation test is a further option to rule out GHD or GHI.^5^

Indeed, in selected individuals the primary radiological examination consists of assessment of skeletal maturation by performing an X-ray of the left hand and wrist for determination of skeletal age. According to Wit et al., the assessment of anatomical abnormalities should be made by the radiologist.^77^ Conversely, a skeletal survey is not appropriate as first-line evaluation but is useful when there is a suspicion of skeletal dysplasia and after consultation with a skeletal radiologist; this investigation highlights typical skeletal features and detects radiographic characteristics suggestive of skeletal dysplasia.

## Genetic testing

After excluding known causes of short stature such as systemic and endocrine diseases, the successive step in the management of a child with ISS is whether to perform genetic testing. In fact, there are multiple causes of short stature or ISS that may not be idiopathic but may be related to unknown genetic mutations. However, genetic testing is not needed for all short children. They should be performed in the diagnostic approach to those children whose clinical features are suspected to have a genetic cause and in whom a clear diagnosis will improve clinical and therapeutic management. Genetic test selection can be directed by a thorough phenotype assessment. In general, individuals with more severe short stature are more likely to have an underlying genetic anomaly. In particular, genetic defects are more likely to be identified in the following conditions: familial forms of isolated GHD or syndromic diseases of multiple pituitary hormone deficiencies, history of consanguinity, severe growth failure (defined as a height <-3 SD for the population or <3 SD than mid-parental target height), body disproportion, skeletal dysplasia and individuals who were small for gestational age and did not achieve sufficient catch-up growth.^76,79,80^

After establishing the need for a genetic test, it is necessary to decide which test is most appropriate. Although a wide range of genetic tests are available, tests identifying a known genetic cause should be used first.

In asymptomatic short girls, the first step is the karyotyping to rule out Turner syndrome. In other cases, the genetic testing approach is debated. If a specific genetic syndrome is suspected, a single genebased test or gene panel is recommended. However, if there is no strong suspicion or if initial testing is normal, the clinician can either accept the diagnosis of ISS or continue with a broader genetic approach to the short child. First, a comparative genomic hybridization array or single nucleotide polymorphism array should be performed to search for copy number variants (microdeletions and microduplications) and uniparental disomies.^81^ Through the use of an array chip, the common single nucleotide polymorphisms are characterized across the genome. This array allows the identification of genomic deletions or duplications related to growth disorders (often syndromic); it also identifies most forms of uniparental disomy, which are not recognized by comparative genomic hybridization array. If no copy number variant is found, the second step is whole exome sequencing using a growth-specific gene panel. This technique sequences the exons (protein-coding portion of the genes) of all coding genes, representing almost 2% of the entire genome and including most of the disease-causing defects. In addition, whole exome sequencing may also identify new genetic variants of unknown aetiological significance. If the genetic test is normal, a methylation analysis should be performed in order to identify methylation disorders in which methyl groups are added to specific nucleotides of the DNA. The methylation suppresses gene transcription and is related to imprinted genes such as in Silver–Russell syndrome.^76^ After confirmation of the mutations, similar tests should be performed in affected and non-affected relatives.^76^

## Conclusions

Despite short stature being one of the most common reasons for consultation with a growth specialist, only a small number of short children receive a molecular diagnosis, and consequently, many children are classified as having ISS. After exclusion of other major and frequent causes of growth failure, clinicians need to evaluate whether a genetic cause might be responsible. In fact, genetic causes of short stature are probably misdiagnosed during clinical practice. One of the main concerns is the lack of a specific correspondence between phenotypic characteristics and possible aetiologies. In these cases, a multiple genetic approach will change the diagnosis of growth failure. The acknowledgment of a genetic aetiology is important for several reasons. Establishing the genetic diagnosis is useful in evaluating the response to GH treatment; it also might guide the hormone therapy, dose administration or type of therapy. Although the choice is mainly dictated by laboratory tests and clinical characteristics, genetic testing becomes useful if the first evaluation has not reached a satisfactory characterization. In addition, it provides some prognostic information, and it may recognize a disorder in which GH treatment is contraindicated. More importantly, the genetic diagnosis offers the advantage of promoting recognition of other family members affected by the same genetic abnormality.

Further research is needed to better characterize children with ISS, evaluating not only the phenotype but also the genotype in order to improve knowledge and apply a translational personalized approach.
